# Bromoditerpenes from the Red Seaweed *Sphaerococcus coronopifolius* as Potential Cytotoxic Agents and Proteasome Inhibitors and Related Mechanisms of Action

**DOI:** 10.3390/md20100652

**Published:** 2022-10-20

**Authors:** Celso Alves, Joana Silva, Susete Pintéus, Romina A. Guedes, Rita C. Guedes, Rebeca Alvariño, Rafaela Freitas, Márcia I. Goettert, Helena Gaspar, Amparo Alfonso, Maria C. Alpoím, Luis M. Botana, Rui Pedrosa

**Affiliations:** 1 MARE—Marine and Environmental Sciences Centre, ARNET—Aquatic Research Network, Politécnico de Leiria, 2520-630 Peniche, Portugal; 2 Research Institute for Medicines (iMed.ULisboa), Faculty of Pharmacy, Universidade de Lisboa, 1649-003 Lisbon, Portugal; 3 Department of Pharmacology, Faculty of Veterinary, University of Santiago de Compostela, 27002 Lugo, Spain; 4 Cell Culture Laboratory, Postgraduate Programme in Biotechnology, University of Vale do Taquari—Univates, CEP 95914-014 Lajeado, RS, Brazil; 5 Department of Pharmaceutical and Medicinal Chemistry, Institute of Pharmacy, Eberhard Karls Universität Tübingen, 72074 Tübingen, Germany; 6 BioISI—Biosystems and Integrative Sciences Institute, Faculty of Science, University of Lisbon, 1749-016 Lisbon, Portugal; 7 Center for Neuroscience and Cell Biology (CNC), University of Coimbra, 3004-517 Coimbra, Portugal; 8 MARE—Marine and Environmental Sciences Centre, ARNET—Aquatic Research Network, ESTM, Politécnico de Leiria, 2520-614 Peniche, Portugal

**Keywords:** terpenes, algae, marine natural products, apoptosis, mitochondrial dysfunction, oxidative stress, anticancer, *Sphaerococcus coronopifolius*

## Abstract

Seaweeds are a great source of compounds with cytotoxic properties with the potential to be used as anticancer agents. This study evaluated the cytotoxic and proteasome inhibitory activities of 12*R*-hydroxy-bromosphaerol, 12*S*-hydroxy-bromosphaerol, and bromosphaerol isolated from *Sphaerococcus coronopifolius*. The cytotoxicity was evaluated on malignant cell lines (A549, CACO-2, HCT-15, MCF-7, NCI-H226, PC-3, SH-SY5Y, and SK-MEL-28) using the MTT and LDH assays. The ability of compounds to stimulate the production of hydrogen peroxide (H_2_O_2_) and to induce mitochondrial dysfunction, the externalization of phosphatidylserine, Caspase-9 activity, and changes in nuclear morphology was also studied on MCF-7 cells. The ability to induce DNA damage was also studied on L929 fibroblasts. The proteasome inhibitory activity was estimated through molecular docking studies. The compounds exhibited IC_50_ values between 15.35 and 53.34 µM. 12*R*-hydroxy-bromosphaerol and 12*S*-hydroxy-bromosphaerol increased the H_2_O_2_ levels on MCF-7 cells, and bromosphaerol induced DNA damage on fibroblasts. All compounds promoted a depolarization of mitochondrial membrane potential, Caspase-9 activity, and nuclear condensation and fragmentation. The compounds have been shown to interact with the chymotrypsin-like catalytic site through molecular docking studies; however, only 12*S*-hydroxy-bromosphaerol evidenced interaction with ALA20 and SER169, key residues of the proteasome catalytic mechanism. Further studies should be outlined to deeply characterize and understand the potential of those bromoditerpenes for anticancer therapeutics.

## 1. Introduction

Marine natural products (MNP) have been revealed to possess uncommon and diverse chemical structures with a great ability to interact with different biological targets, making them excellent candidates to inspire the development of breakthrough pharmacological agents [1,2]. The efforts in discovering new therapeutic entities from marine origin have driven a growing number of marine natural products and derivatives that entered clinical trials, reinforcing their relevance and impact on the pharmaceutical industry [3]. The high success ratio of the approved drugs*/*MNPs reported (1 in 3500 MNPs) compared with the hit rate (1/5000 to 1/10,000) of non-marine-derived NPs indicates their pharmacological relevance [4,5].

Most of the approved MNP-based drugs and compounds currently in clinical trials, due to distinct factors, have an indication for the treatment of malignant tumors, one of the biggest scourges of human health nowadays, potentiated by the COVID-19 pandemic [5,6]. To date, ten MNP-based drugs (out of fourteen) were clinically approved by different health regulatory agencies to treat cancer—nine of them in the last seventeen years [7]. Despite the advances achieved in the last decades, cancer treatments remain a huge challenge due to the multifactorial complexity of malignant diseases [8]. Cancer diseases possess a diverse and complex cellular machinery supported by a microenvironment that sustains proliferative signalling, deregulates cellular metabolism, activates invasion and metastasis, and resists cell death, among other biological events, ensuring the adaptation of malignant cells to therapeutic regimes, developing chemo-resistance [8]. Consequently, the discovery and development of new anticancer therapeutics is of utmost relevance.

In this field, marine natural products have revealed a capacity to modulate distinct cancer intracellular signalling pathways such as cell proliferation, cell viability, endoplasmic reticulum (ER) stress, the induction of reactive oxygen species (ROS) production, apoptosis, etc [9]. Among MNPs, polyphenols, polysaccharides, alkaloids, peptides, and terpenoids have demonstrated great anticancer activities in in vitro, preclinical, and clinical studies [7,10]. Seaweeds represent the third group from which the highest number of new compounds was obtained in recent decades. These belong to distinct chemical classes such as polysaccharides, sterols, phycocyanins, carotenoids, alkaloids, and terpenoids, among others [11]. Those compounds have exhibited a wide range of biological activities including antimicrobial [12], antioxidant [13], anti-inflammatory [14], antiviral [15], and anticancer activities [16]. Regarding anticancer properties, seaweed metabolites have been shown to prevent tumor growth by inducing apoptosis, blocking cell cycle pathways, and affecting the viability of cancer stem cells, which are major players in angiogenesis, invasion, and metastasis [11]. However, the relevance of seaweeds’ bioactive compounds in the treatment of cancer remains underexplored, and their translation to clinical use is not yet a reality.

**Figure 1:**
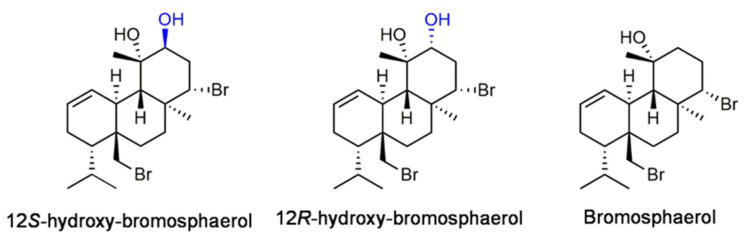
Chemical structures of *Sphaerococcus coronopifolius* bromoditerpenes.

The chemical profile of the red seaweed *Sphaerococcus coronopifolius* Stackhouse 1797 has revealed the presence of structurally diverse diterpenes, many of them brominated, such as 12*R*-hydroxy-bromosphaerol, 12*S*-hydroxy-bromosphaerol, and bromosphaerol (Figure 1), as a consequence of the abundance of halogen ions in the seawater [11,17]. This chemical feature is rare among secondary metabolites and results predominantly from bromine substitution, which seems to enhance their effects on biological systems [18]. Those compounds have displayed different biological activities, including antimicrobial [19], antifouling [20], and cytotoxicity activities [21,22,23]. Regarding the antitumor potential, only preliminary screenings were accomplished, and the mechanism of action underlying their cytotoxic effects remains unclear, as well as their ability to interact with the proteasome. Accordingly, the main goal of this work was to evaluate the cytotoxic activities of those bromoditerpenes on several malignant cell lines and related mechanisms of action, also predicting their ability to act as proteasome inhibitors, as evaluated through molecular docking analysis. The proteasome is essential for the maintenance of the intracellular protein homeostasis, and its dysfunction has been associated with the development of several diseases, such as cancer [24].

The bromoditerpenes displayed moderate cytotoxicity on the studied malignant cell lines, which seems to be mediated by an increase in H_2_O_2_ levels or DNA damage and apoptosis induction.

## 2. Results

### 2.1. Interaction with Proteasome—Molecular Docking Studies

The ability of the three bromoditerpenes (bromosphaerol, 12S-hydroxy-bromosphaerol, and 12*R*-hydroxy-bromosphaerol) to inhibit the 20S proteasome was assessed through molecular docking calculations. The crystal structure with the PDB code 4R67 (human constitutive 20S proteasome in complex with carfilzomib) and the chymotrypsin-like (CT-L) catalytic site composed of the β5 catalytic subunit (and the β6 complementary subunit) were prepared for the docking calculations. The interaction profile showed that the three bromoditerpenes are positioned distantly from the Thr1 N-terminal *γ* hydroxyl group (Thr1O*γ*), which acts as a nucleophile in the catalytic mechanism [25,26,27,28]: bromosphaerol and 12*R*-hydroxy-bromosphaerol are both at a distance of 10.87 Å from the Thr1O*γ*, the 12*S*-hydroxy-bromosphaerol being at 9.26 Å.

**Figure 2:**
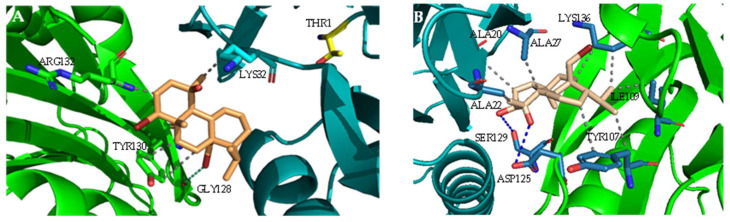

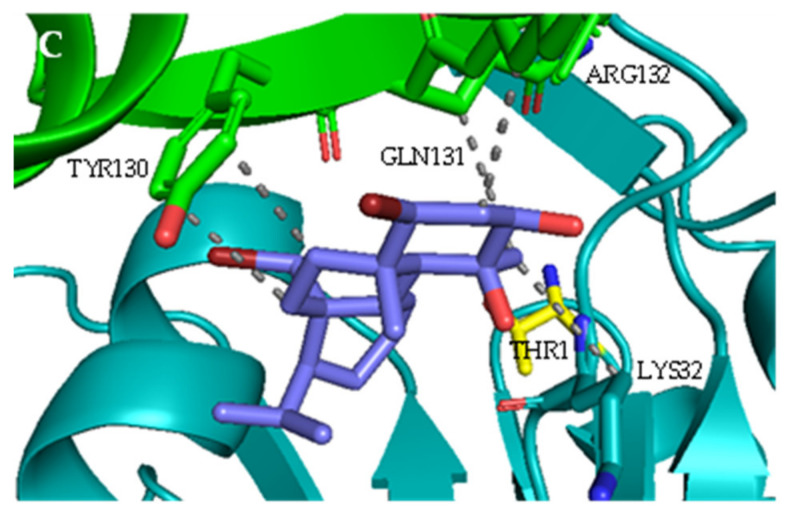
Docking poses of the *Sphaerococcus coronopifolius* bromoditerpenes in the proteasome chymotrypsin-like (CT-L) active site (β5 subunit: cyan; β6 subunit: green; catalytic THR1: yellow). (**A**) Bromosphaerol, (**B**) 12*S*-hydroxy-bromosphaerol, (**C**) 12*R*-hydroxy-bromosphaerol.

The best docking pose for each compound is shown in Figure 2. Bromosphaerol (Figure 2A) interacts with LYS32, TYR130, ARG132, and GLY128 but not with key residues such as THR1, ASP17, ALA20, LYS33, MET45, ALA49, CYS52, SER129, ASP166, and SER169 [29,30,31,32]. The bromoditerpene 12*S*-hydroxy-bromosphaerol (Figure 2B) interacts with ALA20 and SER129, but the remaining interactions are established with non-key residues (ALA22, ALA27, TYR107, ILE109, LYS136, and ASP125). At last, the compound 12*R*-hydroxy-bromosphaerol (Figure 2C) interacts with amino acids that are closer to the key residues of the CT-L activity: LYS32, TYR130, GLN131, and ARG132.

### 2.2. Isolation and Identification of Bromoditerpenes

*Sphaerococcus coronopifolius* freeze-dried samples were subjected to a sequential extraction and fractionation process by vacuum liquid chromatography, resulting in five fractions (F1–F5). The F3 fraction was purified through successive chromatographic techniques, affording three pure compounds. The analysis of their NMR 1D/2D spectra and the comparison of the chemical shifts and structural assignments (Supplementary Material, Tables S1–S3) with the literature data allowed for the unequivocal identification of the purified compounds known as the bromoterpenes 12*R*-hydroxy-bromosphaerol, 12*S*-hydroxy-bromosphaerol, and bromosphaerol (Figure 1).

### 2.3. Cytotoxic Activities on Malignant Cells

**Figure 3:**
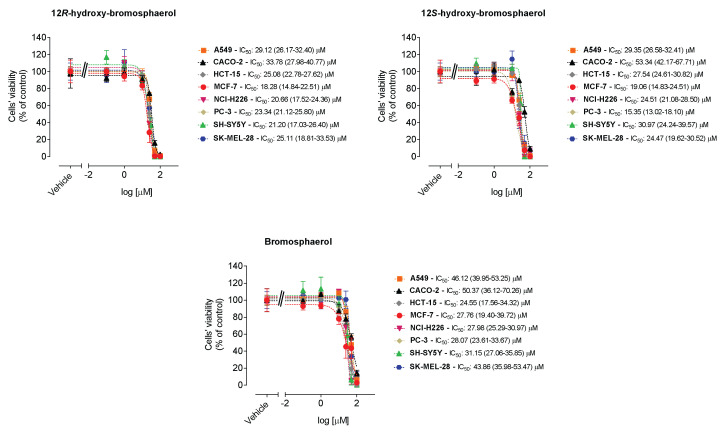
Dose-response analysis (0.1–100 µM) of *Sphaerococcus coronopifolius* bromoditerpenes on malignant cell lines derived from distinct tissues following 24 h of treatment. The effects on cell viability were estimated by the MTT assay. The values in parentheses represent the confidence intervals for 95%.

The cytotoxicity of the compounds was tested on malignant cell lines derived from distinct tissues (A549, CACO-2, HCT-15, MCF-7, NCI-H226, PC-3, SH-SY5Y, and SK-MEL-28), and the results are displayed in Figure 3.

The 12*R*-hydroxy-bromosphaerol and bromosphaerol displayed a range of IC_50_ values between 18.28/27.76 µM and 33.78/50.37 µM, the lowest value observed on MCF-7 cells and the highest one observed on CACO-2 cells, respectively (Figure 3). 12*S*-hydroxybromosphaerol exhibited IC_50_ values between 15.35 µM and 53.34 µM, the smallest value on cells derived from prostate adenocarcinoma (PC-3), and the highest on CACO-2 cells. Overall, the higher IC_50_ values were observed on malignant cells derived from colorectal adenocarcinoma (CACO-2), these being the most resistant to the bromoditerpenes treatment. In some cases, the compounds exhibited effects more marked than those of the anticancer drugs, especially in colorectal cancer cells, when tested in these experimental conditions (Supplementary Material, Figures S1–S3). Since the compounds presented broad anti-cancer activity in several cell lines, further insights into the mechanisms underlying the observed effects were sought. The MCF-7 cell line was used because breast cancer leads the number of diagnoses in women and is the second most deadly cancer after lung cancer [6].

### 2.4. Cytotoxicity and Mitochondrial Function

**Figure 4:**
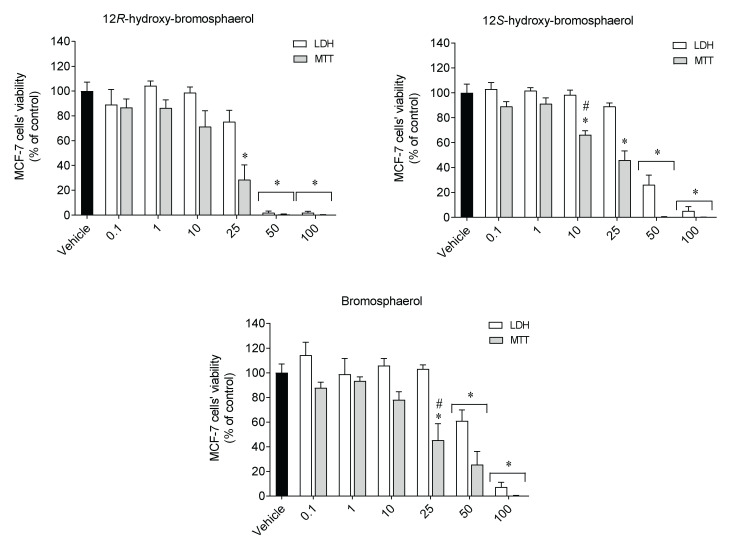
Effects of *Sphaerococcus coronopifolius* bromoditerpenes (0.1–100 µM) on MCF-7 cells’ viability, as estimated by the MTT and LDH assays following exposure for 24 h. Symbols represent significant differences (*p* < 0.05) when compared to the * vehicle and # LDH assay.

To understand if the effects mediated by the compounds were linked to mitochondrial activity, the lactate dehydrogenase (LDH) release was used as an indicator of cell survival, while the mitochondrial function was assessed by the MTT assay. MCF-7 cells were exposed to compounds (0.1*–*100 µM) for 24 h, and the results are presented in Figure 4.

Overall, the MCF-7 cells treated with the compounds revealed a similar profile on both assays; it was more marked on the MTT assay (Figure 4). Through the MTT assay, all compounds at 25 µM showed significant differences when compared to the vehicle treatment. However, this was not observed on the LDH assay. Furthermore, when tested at 10 and 25 µM of 12*S*-hydroxy-bromosphaerol and bromosphaerol, respectively, the results revealed by the MTT assay displayed significant differences when compared with the effects observed on the LDH assay.

### 2.5. Hydrogen Peroxide Levels

**Figure 5:**
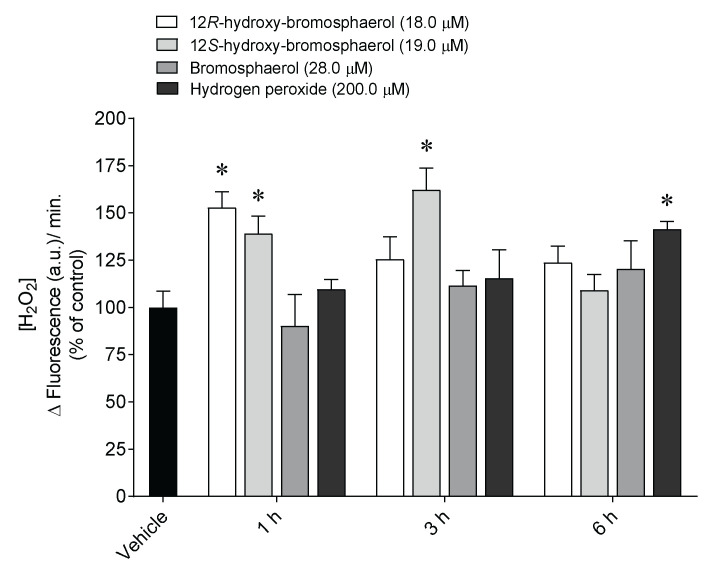
Hydrogen peroxide (H_2_O_2_) levels generated by MCF-7 cells following treatment with *Sphaerococcus coronopifolius* bromoditerpenes (IC_50_) for 1, 3, and 6 h. The symbol represents significant differences (*p* < 0.05) compared to the * vehicle.

Hydrogen peroxide (H_2_O_2_) levels were estimated on MCF-7 cells in real-time following the treatment with the aforementioned compounds (IC_50_) for 1, 3, and 6 h. The results are presented in Figure 5.

The treatment with 12*R*-hydroxy-bromosphaerol and 12*S*-hydroxy-bromosphaerol significantly stimulated the production of hydrogen peroxide (H_2_O_2_) on MCF-7 cells after 1 h of treatment. However, the highest increase was mediated by 12*S*-hydroxy-bromosphaerol on MCF-7 cells after exposure for 3 h. Conversely, bromosphaerol was not able to induce H_2_O_2_ generation.

### 2.6. Depolarization of Mitochondrial Membrane Potential

**Figure 6:**
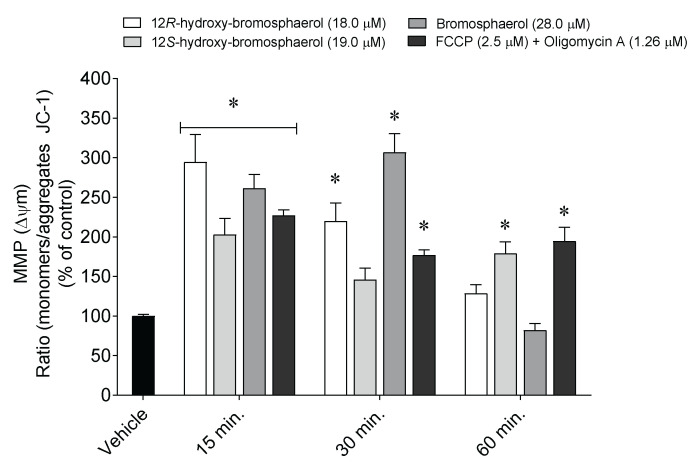
Alterations on the mitochondrial membrane potential of MCF-7 cells following exposure to *Sphaerococcus coronopifolius* bromoditerpenes (IC_50_) for 15, 30, and 60 min. The symbol (*) represents significant differences (*p* < 0.05) compared to the vehicle.

Alterations in the mitochondrial membrane potential were studied following MCF-7 cells’ exposure to the aforementioned compounds (IC_50_) for 15, 30, and 60 min (Figure 6).

The treatment of MCF-7 cells with the isolated bromoditerpenes promoted changes in MMP after 15, 30, and 60 min (Figure 6). 12*R*-hydroxy-bromosphaerol and bromosphaerol induced a significant depolarization after 15 and 30 min, while 12*S*-hydroxy-bromosphaerol induced the same effect after 30 and 60 min as compared to the vehicle situation. However, the highest effect was mediated by 12*R*-hydroxy-bromosphaerol and bromosphaerol after 15 min (294.6 ± 34.7%) and 30 min (306.9 ± 23.5%) of treatment, respectively.

### 2.7. Externalization of Phosphatidylserine, Caspase-9 Activity, and Nuclear Condensation and/or Fragmentation

**Figure 7:**
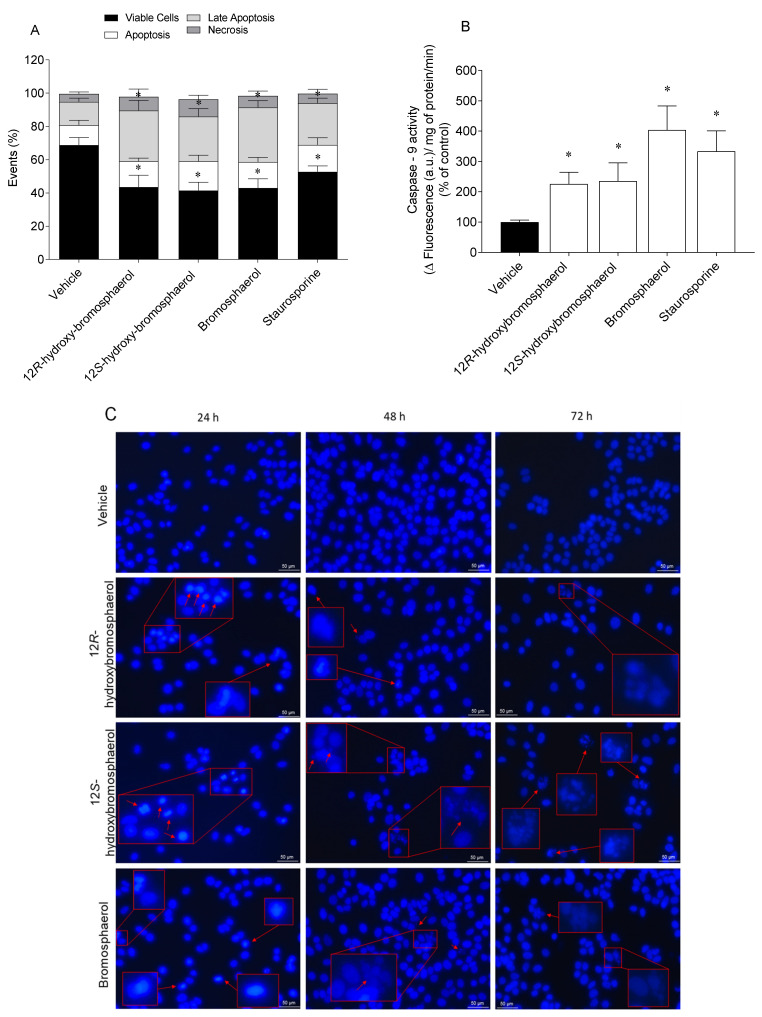
Effects of *Sphaerococcus coronopifolius* bromoditerpenes (IC_50_) on apoptosis biomarkers: (**A**) externalization of phosphatidylserine (24 h), (**B**) Caspase-9 activity (6 h), and (**C**) nuclear morphology (24, 48, and 72 h). The symbol (*) represents significant differences (*p* < 0.05) compared to the vehicle. Images of DAPI-stained cells were acquired using an inverted fluorescence microscope at ×400. Arrows indicate alterations in DNA compared to the vehicle. The images are representative of one well of each tested condition.

Apoptosis is characterized by key biological events, including the externalization of phosphatidylserine, caspases activation, and nuclear condensation and fragmentation. Those events were studied on MCF-7 cells following treatment with the compounds (IC_50_) at different exposition times (Figure 7).

Under controlled conditions, MCF-7 cells were mostly viable, with a small percentage in late apoptosis (Figure 7A). All of the bromoditerpenes promoted a significant decrease in viable cells and cells in the late apoptosis stage compared to the vehicle treatment. As for the apoptosis and necrosis levels, there were no differences between the treated cells and the vehicle. The compounds induced Caspase-9 activity following 6 h exposure, bromosphaerol being the most efficient one (Figure 7B). By analyzing Figure 7C, it is possible to observe changes in MCF-7 cells*’* nuclear morphology. After 24 h and 48 h of exposition to 12*R*-hydroxy-bromosphaerol, nuclear condensation seemed to be induced; however, the effects were much more expressive with 72 h of exposition. 12*S*-hydroxy-bromosphaerol treatment induced nuclear condensation following 24 h of exposure and extensive nuclear fragmentation for prolonged exposures, i.e., 48 h and 72 h. Similarly, bromosphaerol induced nuclear condensation after 24 h and nuclear fragmentation after 48 and 72 h.

### 2.8. DNA Damage on L929 Fibroblasts

**Figure 8:**
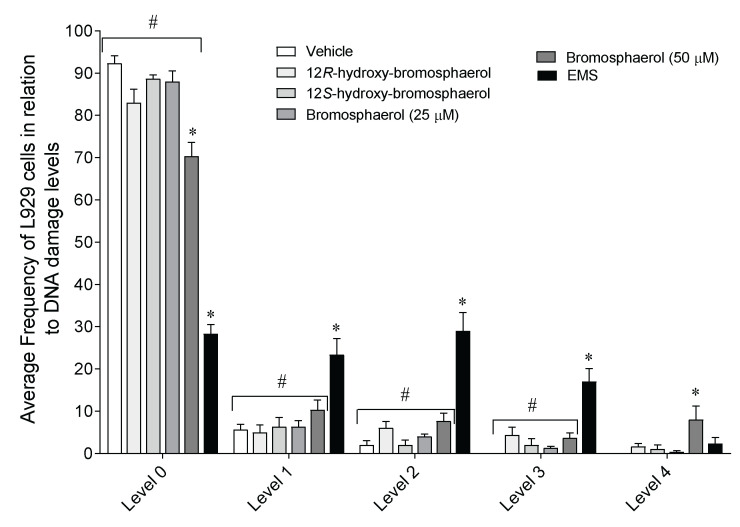
Effects of *Sphaerococcus coronopifolius* bromoditerpenes (25 and/or 50 µM) on the DNA integrity of L929 fibroblasts following 3 h of exposure. Symbols represent significant differences (*p* < 0.05) compared to the * vehicle and # ethyl methanesulfonate (EMS) of the respective DNA damage levels. Damage index: Σ (comet class: 1, 2, 3, 4), 0—nuclei without DNA damage, and 4—nuclei with maximum DNA damage.

The ability of bromoditerpenes (25 and/or 50 µM) to affect the DNA integrity was studied on L929 fibroblasts following 3 h of exposure (Figure 8).

Bromosphaerol and ethyl methanesulfonate (EMS)*—*the positive control for DNA damage—significantly decrease the number of cells at level 0 of the DNA damage index when compared to the vehicle (Figure 8). At levels 1, 2, and 3, only EMS displayed significant differences compared to the vehicle. Particularly, bromosphaerol (50 µM) was the only compound that induced level 4 DNA damage on fibroblasts. However, when tested at 25 µM, this compound exhibited a similar behavior to that of the other tested compounds.

## 3. Discussion

In the last decades, marine natural products have been shown to be key players in the development of innovative anticancer therapeutic strategies, which is easily proved by the currently approved MNP-derived drugs. The ongoing clinical pipeline presents thirty-three marine-derived molecules, from which twenty-seven are being tested for the treatment of different oncologic diseases [7]. Marine natural products display a great ability to act as biological modulators, exhibiting dissimilar modes of action within typical classes of drugs and establishing new avenues for cancer therapy [33,34]. Despite the efforts, the anticancer potential of several MNPs remains poorly explored, evidencing the relevance of performing studies with molecules that are already described but whose mechanisms affecting malignant cell lines are not fully understood. Thus, the present study aimed to evaluate the potential of three bromoditerpenes isolated from the red seaweed *Sphaerococcus coronopifolius* to interact with the proteasome core and induce cytotoxicity on different cancer cell lines, as well as study their possible mechanisms of action.

Proteasome plays a critical role in the regulation of intracellular protein levels, it being responsible for the degradation of most cellular proteins (80%) in the cytoplasm and nucleus after being tagged with ubiquitin. The malfunction of this system can lead to the degradation of normal proteins while the abnormal ones cannot be degraded, resulting in proteasome-related diseases such as cancer [35]. Furthermore, plasma-increased levels of the 20S proteasome catalytic core have been observed in cancer patients, particularly in the case of solid tumors, which can present values that are 1000-fold higher compared with those of healthy individuals [36,37]. These clinical findings suggest that the 20S proteasome is a hot and challenging target in cancer research. Proteasome inhibitors are able to induce apoptosis in several cancer cells, showing low cytotoxicity in normal cells [38]. Regarding the interaction pattern of the three bromoditerpenes with the proteasome CT-L catalytic site, molecular docking calculations showed that the compounds do not interact with the catalytic THR1. However, 12*S*-hydroxy-bromosphaerol interacts with ALA20 and SER169, which are key residues of proteasome catalytic activity. These interactions may be explained by the distinct conformation adopted by the three compounds, suggesting that the presence of an additional hydroxyl group in derivatives of bromosphaerol, as well as the spatial configuration of 12*S* and 12*R*, can be relevant in the interaction. However, considering these results, the interaction pattern of the three compounds, observed through molecular docking studies, is not very likely to lead to effective proteasome inhibition. Similarly, the marine bromoditerpene sphaerococcenol A also did not establish interactions with key amino acids that are essential for recognition and proteasome inhibition [39]. Nevertheless, several other reports attest to the ability of terpenes obtained from plants and marine organisms (e.g., triptolide, celastrol, pristimerin, withaferin A, petrosapongiolide M, and heteronemin) to interact with key residues of proteasome by molecular modelling, which was confirmed by in vitro and in vivo assays [40,41,42,43].

Regarding the cytotoxic effects, the data gathered revealed that those three bromoditerpenes induced a moderate loss of viability in several malignant cell lines with IC_50_ values ranging from 15.35 to 53.34 µM. Regarding A549, PC-3 and SK-MEL-28 tumor cells, the observed IC_50_ range is similar to the IC_50_ values previously reported for those cells (9–35 µM) [23]. By looking to the results attained by the MTT and LDH assays, it is possible to conclude that the effects mediated by 12*S*-hydroxy-bromosphaerol and bromosphaerol were more marked in the MTT assay. Furthermore, despite the treatment with 12*R*-hydroxy-bromosphaerol not promoting significant differences between the assays, the effects on MCF-7 cells’ viability were also more noticeable when estimated by the MTT assay compared to the LDH assay at a concentration near their IC_50_ value (25 µM). Those data are particularly interesting since, although the MTT and LDH assays evaluate the effects on cells’ viability, the cellular target of each method is distinct. In fact, while the MTT assay evaluates the cells’ dehydrogenases activity (both mitochondrial and cytoplasmatic), LDH evaluates the membrane integrity [44]. The results suggest that the compounds may target mitochondrial activity and that their similar effects can be related to the presence of a bromine group in their structures, which is rare in nature and has been associated with their biological activities [18]. Furthermore, 12*R*-hydroxy-bromosphaerol and 12*S*-hydroxy-bromosphaerol are diastereoisomers—specifically, epimers on position 12—which means that the compounds have the same molecular formula and constitutions around the carbon atoms, and the spatial arrangement of the groups around those atoms differ only in terms of the configuration of the stereocenter C12. These spatial differences are enough to mediate distinct pharmacological activities—as observed with thalidomide, a teratogenic drug used in pregnant women in the late 1950s—due to the simultaneous presence of both enantiomers, R and S, in its composition, a fact that was unknown at the time. The (*R*)-enantiomer was responsible for the sedative effects, the therapeutic properties of interest, while the (*S*)-isomer induced teratogenic activities, provoking a range of severe birth defects [45]. However, despite 12*R*-hydroxy-bromosphaerol and 12*S*-hydroxy-bromosphaerol displaying different spatial arrangements around C12, this seemed to not influence the cytotoxic activities, since those compounds exhibited similar activities on all cell lines.

Apoptosis has been a mainstay and a goal of innovative oncology therapeutics to ensure an effective elimination of cancer cells. Apoptotic signaling pathways are triggered by multiple factors, including cellular stress and DNA damage [46]. Several chemotherapeutics, such as cisplatin and doxorubicin, can stimulate the formation of reactive oxygen species (ROS), leading to cancer cells’ death by apoptosis [47] and demonstrating that the induction of ROS production could be an appropriate therapeutic strategy. On the other hand, the mechanism of action of other anticancer drugs is associated with the induction of high levels of DNA damage that trigger cell cycle checkpoints, leading to cell cycle arrest and/or cell death [48]. This strategy is based on two fundamental assumptions. The first relies on the fact that cancer cells multiply more often than normal cells; secondly, normal cells have a highly efficient DNA damage response and DNA repair mechanisms, giving normal cells the ability to stop the cell cycle to repair the DNA. Therefore, they have the ability to arrest proliferation for more efficient DNA repair and to avoid the damage mediated by the drug [48,49]. Herein, the ability of compounds to stimulate the production of hydrogen peroxide (H_2_O_2_) and DNA damage was studied, as well as the capacity to modulate different biomarkers related to apoptosis. The compounds 12*R*-hydroxy-bromosphaerol and 12*S*-hydroxy-bromosphaerol stimulated the production of H_2_O_2_, and bromosphaerol induced DNA damage, suggesting that the presence of an additional hydroxyl group in the derivatives 12*S* and 12*R* induces a distinct effect between the derivatives and bromosphaerol. These effects were accompanied by changes in mitochondrial membrane potential, more cells in late apoptosis, increased Caspase-9 activity, and nuclear fragmentation/condensation. The ability of marine terpenes to modulate these intracellular signaling pathways has already been reported. The marine sesterterpenoid heteronemin isolated from the sponge *Kyrgios* sp. induced apoptosis and ferroptosis triggered by the production of ROS on several in vitro cancer cellular models [50,51]. Furthermore, previous studies conducted with the bromoditerpenes sphaerococcenol A and sphaerodactylomelol also displayed increased H_2_O_2_ levels, modulating different apoptotic hallmarks [39,52]. However, further studies need to be accomplished to fully understand the involvement of oxidative stress in MCF-7 cells’ death and loss of cell viability. For instance, a pre-treatment with N-acetylcysteine (NAC), known as an ROS scavenging molecule, can be used to understand if the cytotoxic effects mediated by 12*R*-hydroxy-bromosphaerol and 12*S*-hydroxy-bromosphaerol are related to the increase in H_2_O_2_ levels. Despite the mechanisms of action underlying the activities of these compounds being unexplored, a potential cytostatic effect of 12*S*-hydroxy-bromosphaerol was suggested on U373 GBM cells. Smyrniotopoulos and co-workers [23] observed a marked increase in mitosis length through the computer-assisted phase-contrast microscopy approach. Accordingly, this evidence and our data suggest that 12*S*-hydroxy-bromosphaerol may have the capacity to arrest the cell cycle and trigger apoptosis, two independent physiological processes regulated by a complex network of pathways, including cell cycle regulators, such as P53, that are essential for tissue homeostasis [53].

On the other hand, according to the findings observed following bromosphaerol treatment, it is possible to suppose that its effects may be associated with the induction of DNA damage, leading to the activation of cell death by apoptosis. Among the tested compounds, bromosphaerol was the only one that induced significant DNA damage on L929 cells. However, when tested at a similar concentration (25 µM, the IC_50_ value for MCF-7 cells, 3 h), L929 cells were not affected. However, it is possible that the longer treatment of MCF-7 cells triggered DNA damage repair mechanisms, leading to cells’ survival by recovering the effects mediated by bromosphaerol observed on L929 cells after 3 h. Nevertheless, more studies are needed to validate the different hypotheses, including the evaluation of the genotoxic effects over time on L929 cells, carrying out those assays on MCF-7 cells, as well as studying the cell cycle. Although several NPs induce DNA damage, leading to cell cycle arrest and apoptosis [54,55,56], the present study discloses, for the first time, the potential mechanisms of action underlying the cytotoxic effects of the marine bromoditerpenes 12*R*-hydroxy-bromosphaerol, 12*S*-hydroxy-bromosphaerol, and bromosphaerol, which may be related to increased H_2_O_2_ levels and DNA damage, leading to cell death by apoptosis. Further experiments are required to understand the involvement of oxidative stress and DNA damage in the cytotoxic activity of these compounds, as well as studies on their effects on normal cells (proliferating or quiescent) in order to define their therapeutic index and understand their antitumor potential.

## 4. Materials and Methods

### 4.1. Collection, Extraction, and Purification

Red seaweed specimens of *Sphaerococcus coronopifolius* were collected in the Berlengas Nature Reserve (39°24′44.8′′ N 9°30′29.5′′ W), Peniche, Portugal by scuba diving and immediately transported to MARE-Polytechnic of the Leiria laboratory, where they were identified by Prof. Teresa Mouga, a biologist with vast experience in the taxonomic identification and ecology of marine seaweeds. The samples were cleaned to remove detritus, sand, and epibionts and freeze-dried. The dry algal material was powdered and then sequentially extracted with methanol and dichloromethane (1:4) for 12 h. The dichloromethane extract was concentrated to dryness in a rotary evaporator and subjected to normal phase vacuum liquid chromatography on silica gel 60 (0.06–0.2 mm), using cyclohexane (VWR, Fontenay-sous-bois, France) with increasing amounts (25%) of ethyl acetate (EtOAc) (VWR, Fontenay-sous-bois, France) as the mobile phase (five fractions). Fraction F3 was purified by the semi-preparative reversed-phase HPLC column (Synergi Fusion-RP 80 Å, Phenomenex, 10 × 250 mm, 4 μm) at a flow rate of 5.19 mL/min (25 °C), using a mixture of H_2_O: CH_3_CN (VWR, Fontenay-sous-bois, France) as a mobile phase in isocratic conditions from 0 to 5 min (25:75), a linear gradient from 5 to 25 min (from 25:75 to 15:85), and isocratic after 25 min (15: 85). This first purification step afforded 12 sub-fractions (P1–P12), from which only bromosphaerol (P7; tR 23.4 min) was isolated. The sub-fraction P1 was purified on a silica gel 60 column (Sharlau, 0.04–0.06 mm; column height: 19.5 cm; diameter: 2 cm; collection volume: 10 mL) using a mixture of EtOAc/n-Hex (1:9) as a mobile phase, affording 12*S*-hydroxy-bromosphaerol (P1.1, f12 to f15). The sub-fraction P2 was purified by repeating the chromatographic steps: first, by the semi-preparative reversed-phase HPLC column (Synergi Fusion-RP 80 Å, Phenomenex, 10 x 250 mm, 4 μm) using isocratic conditions (60% CH_3_CN: 40% H_2_O) at a flow rate of 5.0 mL/min (25 °C), affording seven sub-fractions (P2.1–P2.7). Fraction P2.5 was then subjected to preparative column chromatography with silica gel 60 (Sharlau, 0.04–0.06 mm; column height: 19.0 cm; diameter: 2 cm; collection volume: 10 mL) eluted with a mixture of *n*-Hex/EtOAc (8:2) along the first 20 fractions, followed by elution with n-Hex/EtOAc (3:7). This step afforded 12*R*-hydroxy-bromosphaerol (f5 to f9 fraction). The structural elucidation of compounds was attained by NMR spectroscopy analysis, 1D (^1^H and ^13^C Attached Proton Test (APT)) and 2D (COSY, HMBC, HSQC-ed) techniques, and by comparison with previously reported data. For biological evaluation, the compounds were dissolved in DMSO (concentration below 0.2%) and evaluated through a set of in vitro assays. The control situation was always treated with the highest concentration of DMSO as the vehicle.

### 4.2. In Silico Docking Studies

The receptor structure was retrieved from the Protein Data Bank (www.rcsb.org) through the PDB code 4R67 (Human constitutive 20S proteasome in complex with carfilzomib, 2.89 Å) [32]. To prepare the protein structure for docking calculations, all atoms (i.e., ligand, salts, water, other chains) other than the receptor β5 and β6 subunits (chains L and M, respectively—CT-L active site) were deleted from the X-ray structure using the MOE software package (v.2019.0102) (Molecular Operating Environment; Chemical Computing Group ULC, 1010 Sherbrooke St. West, Suite #910, Montreal, QC, Canada, H3A 2R7, 2021). The AMBER99 forcefield was used to assign atom types and charges to each atom in the receptor. Hydrogen atoms were added, and the appropriate protonation states were assigned using the Protonate-3D tool within the MOE software package (pH 7.4 and T = 310 K). Structures of bromoditerpenes were built and energy-minimized using MOE. Molecular docking simulations were performed using GOLD 5.4 software [43]. The binding site was defined to be centered in the catalytic Thr1Oγ (β5 catalytic subunit) with a 15 Å search radius. Noncovalent docking calculations were performed, with the number of genetic algorithm (GA) runs set to 1000 and the search efficiency set to 100%, and the ten top-ranked solutions were selected. For the other settings, the default parameters were used. First, an initial docking validation step was carried out by performing self-docking calculations using GOLD (scoring functions: GoldScore, Chemscore, ChemPLP, and ASP) and 1000 exhaustive search runs. All protein amino acid residues were kept rigid, whereas all single bonds of the ligands were treated as fully flexible. The docking parameters (scoring function—ChemPLP—and protein 3D structure) selected were able to successfully reproduce the experimental pose (RMSD < 2 Å between the experimental and predicted pose). The docking procedure was subsequently used for the docking calculations. For the analysis of protein–ligand interactions, the top docking poses were submitted to the detection of residue contacts using the docker implementation of PLIP [57]. Images of the compound and the PDB structures were produced using PyMOL v.1.8.4.0.

### 4.3. Cell Culture Conditions

The cell lines were obtained from the DSMZ and ATCC biobanks. A549 (ATCC: CCL-185) and SH-SY5Y (ATCC: CRL-2266) cells were cultivated in DMEM/F-12 medium (Merck, Germany) supplemented with 10% serum bovine fetal (FBS) (Gibco, USA), GlutaMAX™ (Gibco, USA), 100 IU/mL penicillin, and 100 μg/mL streptomycin (Sigma, USA). CACO-2 (DSMZ: ACC 169), HCT-15 (DSMZ: ACC 357), L929 (DSMZ: ACC 2), MCF-7 (DSMZ: ACC 115), NCI-H226 (Lung squamous cell carcinoma; ATCC: CRL-5826), and PC-3 (Prostate adenocarcinoma; ATCC: CRL-1435) were cultured in RPMI medium supplemented with 10% FBS, 100 IU/mL penicillin, and 100 μg/mL streptomycin. SK-MEL-28 cells (ATCC: HTB-72) were grown in EMEM medium (Sigma, USA) supplemented with 10% FBS, 100 IU/mL penicillin, and 100 μg/mL streptomycin. The cells were dissociated with trypsin (Sigma-Aldrich, USA) and then neutralized with the respective fresh medium and centrifuged at 290 g for 5 min at room temperature. After that, the cells were resuspended in a fresh medium (1:10), seeded in 25 cm^2^ T-Flasks, and cultivated in 5% CO_2_ and a humidified atmosphere at 37 °C.

### 4.4. Cytotoxicity Assays

The cells were seeded in 96-well plates (A549: 2.5×10^5^ cells/mL; CACO-2: 2.5×10^5^ cells/mL; HCT-15: 2.5×10^5^ cells/mL; MCF-7: 2.5×10^5^ cells/mL; NCI-H226: 7.5×10^4^ cells/mL; PC-3: 1.25×10^5^ cells/mL; SH-SY5Y: 2.5×10^5^ cells/mL; SK-ML-28: 2.5×10^5^ cells/mL; AML-12: 5.0×10^4^ cell/mL). After 24 h of seeding, the cancer cell lines were exposed to 12*S*-hydroxy-bromosphaerol, 12*R*-hydroxy-bromospherol, and bromosphaerol (0.1–100 µM) for 24 h, and the effects were assessed by the 3-(4,5-dimethylthiazol-2-yl)-2,5-diphenyltetrazolium bromide (MTT) assay [22]. Cisplatin, tamoxifen, and 5-fluorouracil (all from Sigma, Shanghai, China) were used as anticancer standard controls (0.1–500 µM; 24 h). The cell death was measured by the LDH cytotoxicity assay kit (Pierce™ LDH Cytotoxicity Assay Kit; ThermoScientifc, Rockford, USA) according to the manufacturer’s instructions. Saponin (Sigma, Darmstadt, Germany) (0.4 mg/mL) was used as the positive control for cell death, reducing the cell viability in 100%.

### 4.5. Hydrogen Peroxide Levels

The levels of hydrogen peroxide (H_2_O_2_) were estimated in MCF-7 cells (2.5×10^5^ cells/mL) using the Amplex™ Red hydrogen peroxide assay kit (Molecular probes, Eugene, OR, USA), following treatment with the compounds IC_50_ concentration and H_2_O_2_ (200.0 µM) for 1, 3, and 6 h.

### 4.6. Mitochondrial Membrane Depolarization

MCF-7 cells (2.5×10^5^ cells/mL) were seeded in 96-well plates and treated with the compounds at the IC_50_ concentration and FCCP (2.5 µM) (Sigma, Rehovot, Israel) plus oligomycin A (1.26 µM) (Sigma, St. Louis, MO, USA) conjugate solution for 15, 30, and 60 min. The effects were estimated using the JC-1 (Molecular Probes, Eugene, OR, USA) fluorescent probe [58] through the measurement of JC-1 aggregates (λ excitation: 490 nm; λ emission: 590 nm) and monomers (λ excitation: 490 nm; λ emission: 530 nm) using a plate reader (Bio-Tek Synergy plate reader, Bedfordshire, UK).

### 4.7. Translocation of Phosphatidylserine and Membrane Integrity

MCF-7 cells (1.0×10^6^ cells/mL) were seeded in 6-well plates and treated with the compounds at the IC_50_ concentration and staurosporine (1 µg/mL) (Sigma, Rehovot, Israel) for 24 h, and the effects were analyzed by flow cytometry using the Apoptosis Detection Kit (Immunostep, Salamanca, Spain). Ten thousand events were recorded with the AMNIS imaging flow cytometer using the AMNIS INSPIRE™ software. The data were analyzed using the AMNIS IDEAS™ software (Amnis Corporation v6.0, Luminex Corp, Austin, TX, USA).

### 4.8. Caspase-9 Activity

Enzyme activity was estimated in MCF-7 cells (1.0×10^6^ cells/mL) previously seeded in 6-well plates following a 6 h treatment with the compounds at the IC_50_ concentration and staurosporine (1 µg/mL). The Caspase 9 Fluorimetric Assay Kit (Biovision, Milpitas, CA, USA) was used.

### 4.9. DAPI Staining

DNA nuclear morphology was evaluated using the DAPI fluorescent probe [58]. MCF-7 cells (1.0×10^6^ cells/mL) were seeded in 6-well plates and treated with compounds at the IC_50_ concentration for 24, 48, and 72 h. DNA morphology was analyzed using a fluorescence inverted microscope (ZEISS Axio, VERT. A1, equipped with an AxioCam MRC-ZEISS camera, München, Germany), and a representative image of each treatment was presented.

### 4.10. DNA Damage

L929 mouse fibroblasts (2.0×10^4^ cells/mL) were seeded in 12-well plates and cultured overnight. The fibroblasts were then treated with compounds (25 and/or 50 µM) and ethyl methanesulfonate (200 µg/mL) as a positive control for 3 h. The DNA damage was determined according to the protocol established by Singh et al. [59], with slight modifications [52]. One-hundred cells were randomly chosen, analyzed, visually scored, and classified into five levels according to the tail size formed by breaks in the DNA. Non-overlapping was performed.

### 4.11. Data and Statistical Analysis

At least three independent experiments were carried out in triplicate, and the results were presented as the mean ± standard error of the mean (SEM) and the half-maximal inhibitory concentration (IC_50_). ANOVA with Dunnett’s multiple comparison of group means analysis was accomplished, and the Tukey’s test was applied for multiple comparisons. When applicable, Student’s *t*-test was used. Differences were considered significant at the level of 0.05 (*p* < 0.05). The IBM SPSS Statistics 24 (IBM Corporation, Armonk, NY, USA) and GraphPad v5.1 (GraphPad Software, La Jolla, CA, USA) software were used to accomplish the analysis.

## Data Availability

The data presented in this study are available on request from the corresponding author.

## Supplementary Materials

The following supporting information can be downloaded at: www.mdpi.com/article/10.3390/md20100652/s1, Figure S1: Dose-response curves of cisplatin (0.1–250 µM; 24 h) on malignant cells’ viability (% of control) for IC_50_ determination; Figure S2: Dose-response curve of tamoxifen (0.1–100 µM; 24 h) on MCF-7 cells’ viability (% of control) for IC_50_ determination; Figure S3: Dose-response curve of 5-fluorouracil (0.1–500 µM; 24 h) on colorectal cancer cells’ viability (% of control) for IC_50_ determination; Table S1: ^1^H (400 MHz) and ^13^C (125 MHz) NMR data (CDCl_3_) of bromosphaerol; Table S2: ^1^H (400 MHz) and ^13^C (125 MHz) NMR data (CDCl_3_) of 12*S*-hydroxy-bromosphaerol; Table S3: ^1^H (400 MHz) and ^13^C (125 MHz) NMR data (CDCl_3_) of 12*R*-hydroxy-bromosphaerol. References [21,60] are cited in the Supplementary Materials.
