# Supplementary material for: Bromoditerpenes from the Red Seaweed Sphaerococcus coronopifolius as Potential Cytotoxic Agents and Proteasome Inhibitors and Related Mechanisms of Action
Source: Mar Drugs. 2022 Oct 20;20(10):652. doi: 10.3390/md20100652 (PMC9604732; doi:10.3390/md20100652)
Supplement: Supplementary file 1 [file marinedrugs-20-00652-s001.zip › marinedrugs-1874918-supplementary.pdf]

marine drugs

Supplementary Materials

Bromoditerpenes from the red seaweed *Sphaerococcus coronopifolius* as potential cytotoxic agents and proteasome inhibitors and related mechanisms of action

- **NMR Data**

Table S1. ^{1}H (400 MHz) and ^{13}C (125 MHz) NMR data ($CDCl_3$) of bromosphaerol.

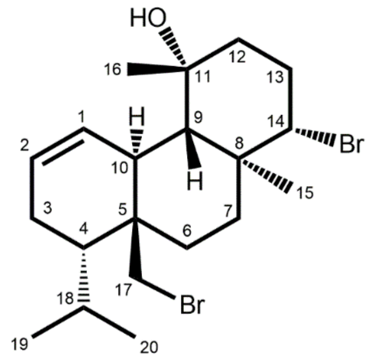

Atom n°	Tested compound δ^1H, *m*, *J* (Hz)	δ^{13}C	Literature Data[1] δ^1H, *m*, *J* (Hz)	δ^{13}C	Group
1	6.01 *br d* (10.6)	128.59	6.01 *br d* (10.5)	128.65	CH
2	5.69 *m* (10.6)	126.96	5.69 *dm* (10.5)	126.88	CH
3	2.14 *m*<br>1.93 *m*	21.88	2.13 *m*<br>1.92 – 2.00 *m*	21.91	CH_2
4	1.76 *m*	42.56	1.65 – 1.88 *m*	42.64	CH
5	-	40.72	-	40.76	C
6	1.81 *m*<br>1.52 *m*	24.97[3]	1.28 *m*<br>1.65 – 1.88 *m*	36.52[2]	CH_2
7	1.85 *m*<br>1.25 *m*	36.44[3]	1.65 – 1.88 *m*<br>1.46 – 1.54 *m*	25.04[2]	CH_2
8	-	41.87	-	41.89	C
9	1.48 *d* (10.9)	50.60	1.46 – 1.54 *m*	50.67	CH
10	2.98 *dm* (10.9)	37.34	2.97 *br d* (10.2)	37.41	CH
11	-	72.31	-	72.75	C
12	1.58-1.68 *m*	46.21	1.65 – 1.88 *m*	46.18	CH_2
13	2.41 *qd* (13.0, 4.4)	30.10	2.41 *ddd* (13.3, 12.4, 4.1)<br>1.92 – 2.00 *m*	30.16	CH_2
14	3.99 *dd* (12.5, 3.5)	68.82	4.00 dd (12.4, 3.4)	68.80	CH
15	1.30 *s*	14.02	1.30 *s*	14.07	CH_3
16	1.38 *s*	35.04	1.38 *s*	35.02	CH_3
17	3.61 *brd* (10.5)<br>3.93 *d* (10.5)	40.37	3.61 d (10.5)<br>3.93 d (10.5)	40.36	CH_2
18	1.95 *m*	25.85	1.92 – 2.00 *m*	25.88	CH
19*	0.90 *d* (6.9)	19.84	0.90 *d* (6.8)	19.78	CH_3
20*	0.97 *d* (6.8)	26.05[4]	0.97 *d* (6.8)	20.01	CH_3

[1] NMR data (500 MHz, $CDCl_3$) from De Rosa *et al.* [60]; [2] The carbones C6 and C7 were incorrectly assigned in De Rosa *et al.* [60]; [3] Correct assignment of C6/C7 based on $^{13}C/^{1}H$ correlations observed in HMBC spectrum (1.30s/36.44 H-15/C-7; 3.93d/24.97 H-17/C-6).

marine drugs

Table S2 - ^{1}H (400 MHz) and ^{13}C (125 MHz) NMR data ($CDCl_3$) of 12*S*-hydroxy-bromosphaerol.

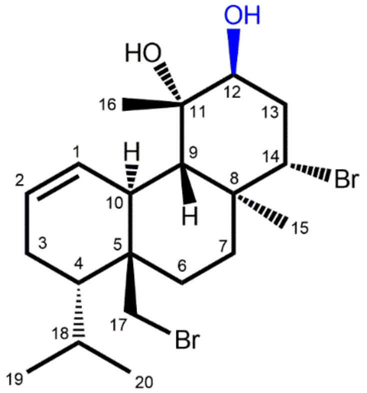

Atom n°	Tested compound δ ^{1}H, *m*, *J* (Hz)	δ ^{13}C	Literature Data[1] δ ^{1}H, *m*, *J* (Hz)	δ ^{13}C	Group
1	5.99 *brd* (10.6)	128.46	5.97 *br d* (10.4)	128.5	CH
2	5.71 *m*	127.54	5.69 *ddt* (10.4, 5.0, 2.5)	127.5	CH
3	1.95 *m*<br>2.14 *m*	21.93	α 1.93 *m*<br>β 2.10 *m*	21.9	CH_2
4	1.74 *m*	42.62	1.72 *br s*	42.6	CH
5	-	41.07	-	41.1	C
6	1.80 *m*<br>1.53 *m*	24.87	α 1.75 *m*<br>β 1.51 *m*	24.9	CH_2
7	1.84 *m*<br>1.31 *m*	36.42	α 1.81 *m*<br>β 1.29 *m*	36.4	CH_2
8	-	41.78	-	41.8	C
9	1.80 *m*	45.95	1.78 *m*	45.9	CH
10	2.99 *dm* (11.2)	36.82	2.97 *dm* (11.2)	36.8	CH
11	-	74.83	-	74.8	C
12	3.47 *m*	79.41	3.45 *br s*	79.4	CH
13	2.16 *dt* (13.8, 3.7)<br>2.72 *td* (13.7, 2.7)	37.38	β 2.14 *dt* (13.7, 3.7)<br>α 2.70 *ddd* (13.7, 12.8, 2.9)	37.4	CH_2
14	4.49 *dd* (12.6, 3.8)	63.36	4.46 *dd* (12.8, 3.7)	63.4	CH
15	1.29 *s*	14.87	1.27 *s*	14.9	CH_3
16	1.46 *s*	31.84	1.44 *s*	31.8	CH_3
17	3.62 *dd* (10.6, 1.7)<br>3.95 *d* (10.6)	40.54	3.60 *dd* (10.8, 2.1)<br>3.93 *d* (10.8)	40.5	CH_2
18	1.96 *m*	25.90	1.93 *m*	25.9	CH
19	0.91 *d* (6.8)	19.71	0.89 *d* (7.0)	19.7	CH_3
20	0.97 *d* (6.8)	25.93	0.95 *d* (7.0)	25.9	CH_3
OH					OH

[1] NMR data (500 MHz, $CDCl_3$) from Smyrniotopoulos *et al*. [21].

marine drugs

Table S3. ^{1}H (400 MHz) and ^{13}C (125 MHz) NMR data ($CDCl_3$) of 12*R*-hydroxy-bromosphaerol.

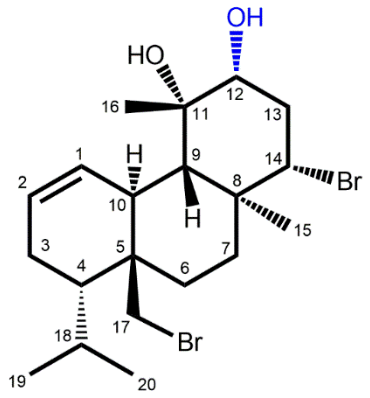

	Tested compound		Literature Data [1]		
**Atom n°**	**δ^{1}H, *m*, *J* (Hz)**	**δ^{13}C**	**δ^{1}H, *m*, *J* (Hz)**	**δ^{13}C**	**Group**
1	6.01 *br d* (10.6)	128.79	5.99 *br d* (10.4)	128.8	CH
2	5.69 *m*	126.41	5.67 *ddt* (10.4, 5.8, 2.9)	126.4	CH
3	2.14 *m*<br>1.93 *m*	21.80	β: 2.12 *m*<br>α: 1.91 *m*	21.8	CH_2
4	1.77 *m*	42.63	1.73 *m*	42.5	CH
5	-	40.47	-	40.5	C
6	1.81 *m*<br>1.52 *m*	24.94	α: 1.77 *m*<br>β 1.50 *m*	24.9	CH_2
7	1.82 *m*<br>1.22 *m*	35.97	α: 1.78 *m*<br>β: 1.18 *m*	36.0	CH_2
8	-	41.97	-	42.0	C
9	1.40 *d* (10.7)	48.71	1.38 *d* (10.8)	48.7	CH
10	3.03 *dm* (10.7)	37.49	3.02 *dm* (10.8)	37.5	CH
11	-	73.46	-	73.5	C
12	3.37 *dd* (11.7, 5.2)	76.94	3.34 *dt* (11.6, 5.4)	76.9	CH
13	2.23 *ddd* (12.4, 5.2, 3.2)<br>2.35 *q* (12.4)	38.03	β 2.21 *ddd* (12.4, 5.4, 3.3)<br>α: 2.33 *ddd* (12.8, 12.4, 11.6)	37.9	CH_2
14	3.90 *dd* (12.8, 3.2)	62.96	3.88 *dd* (12.8, 3.3)	63.0	CH
15	1.26 *s*	13.64	1.25 *s*	13.6	CH_3
16	1.43 *s*	30.86	1.41 *s*	30.9	CH_3
17	3.91 *d* (10.3)<br>3.60 brd (10.3)	40.28	3.89 *d* (10.4)<br>3.59 *dd* (10.4, 1.7)	40.3	CH_2
18	1.94 *m*	25.83	1.93 *m*	25.8	CH
19*	0.91 *d* (6.8)	19.94	0.89 *d* (7.1)	19.9	CH_3
20*	0.98 *d* (6.8)	26.13	0.95 *d* (7.1)	26.1	CH_3

[1] NMR data (500 MHz, $CDCl_3$) from Smyrniotopoulos *et al.* [21]

marine drugs

MDPI

- Cytotoxic activities of anticancer standard drugs

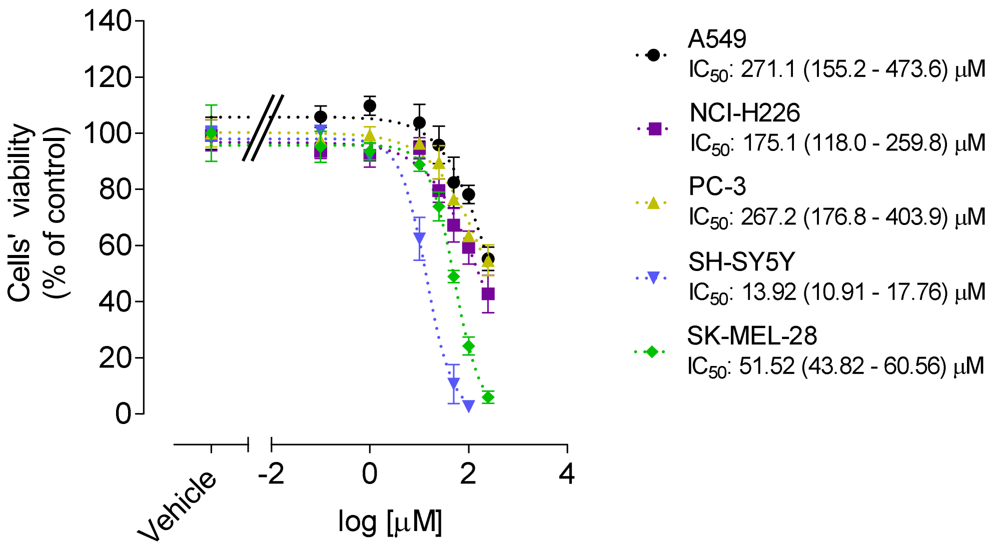

Figure S1. Dose-response curves of cisplatin (0.1 - 250 µM; 24 h) on malignant cells' viability (% of control) for IC_{50} determination. The effects were revealed through the MTT assay. The values represent mean ± SEM of at least three independent experiments carried out in triplicate. The values in parentheses represent the confidence intervals for 95%.

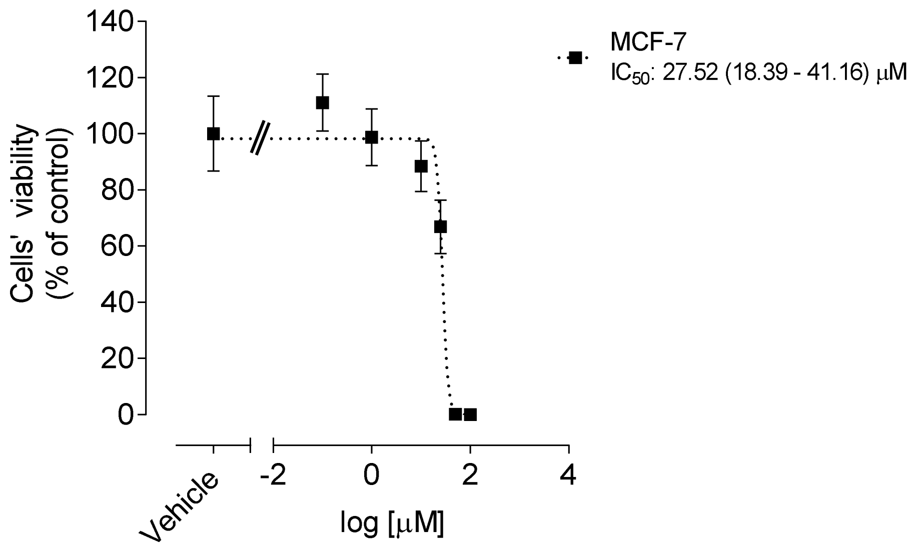

Figure S2. Dose-response curve of tamoxifen (0.1 - 100 µM; 24 h) on MCF-7 cells' viability (% of control) for IC_{50} determination. The effects were revealed through the MTT assay. The values represent mean ± SEM of at least three independent experiments carried out in triplicate. The values in parentheses represent the confidence intervals for 95%.

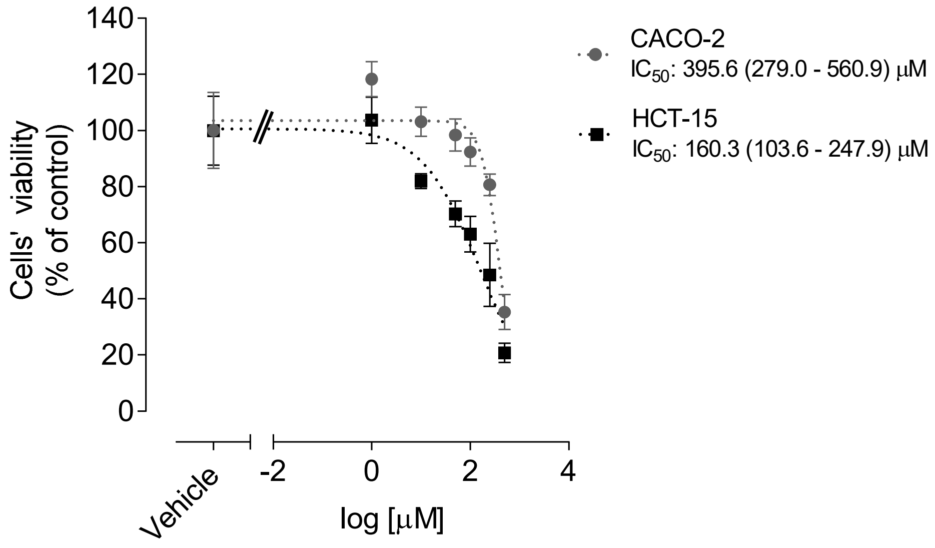

Figure S3. Dose-response curve of 5-fluorouracil (0.1 – 500 μM; 24 h) on colorectal cancer cells' viability (% of control) for IC_{50} determination. The effects were revealed through the MTT assay. The values represent mean ± SEM of at least three independent experiments carried out in triplicate. The values in parentheses represent the confidence intervals for 95%.
